# Identification and Effect Decomposition of Risk Factors for *Brucella* Contamination of Raw Whole Milk in China

**DOI:** 10.1371/journal.pone.0068230

**Published:** 2013-07-10

**Authors:** Pengbo Ning, Mancai Guo, Kangkang Guo, Lei Xu, Min Ren, Yuanyuan Cheng, Yanming Zhang

**Affiliations:** 1 College of Veterinary Medicine, Northwest A&F University, Yangling, Shaanxi, PR China; 2 College of Science, Northwest A&F University, Yangling, Shaanxi, PR China; Iowa State University, United States of America

## Abstract

**Background:**

Lack of clear risk factor identification is the main reason for the persistence of brucellosis infection in the Chinese population, and there has been little assessment of the factors contributing to *Brucella* contamination of raw whole milk. The purpose of this study was to identify risk factors affecting *Brucella* contamination of raw milk, and to evaluate effective measures for disease reduction in order to determine preventive strategies.

**Methods and Findings:**

A nationwide survey was conducted and samples were obtained from 5211 cows corresponding to 25 sampling locations throughout 15 provinces in China. The prevalence of *Brucella* in the raw milk samples averaged 1.07% over the 15 Chinese provinces, while the prevalence of positive areas within these regions ranged from 0.23–3.84% among the nine provinces with positive samples. The survey examined factors that supposedly influence *Brucella* contamination of raw whole milk, such as management style, herd size, abortion rate, hygiene and disease control practices. A binary logistic regression analysis was carried out to determine the association between risk factors for *Brucella* and contamination of milk samples. Furthermore, a relative effect decomposition study was conducted to determine effective strategies for reducing the risk of *Brucella* contamination of raw whole milk. Our data indicate that disease prevention and control measures, abortion rate, and animal polyculture are the most important risk factors. Meanwhile, culling after quarantine was identified as an effective protective measure in the current Chinese dairy situation.

**Conclusions:**

These results indicate that, although there is a low risk of contamination of milk with *Brucella* nationwide in China, there are individual regions where contamination is a significant problem. Controlling three factors–culling after quarantine, maintaining a low abortion rate, and avoiding mixing groups of cattle and small ruminants–could effectively reduce the risk of *Brucella* contamination of raw whole milk.

## Introduction

Brucellosis is a zoonotic bacterial infection that is a serious public health problem in many countries of the world [1]. It results in decreased animal production and large economic losses as a consequence of abortion, sterility, decreased milk production, reduced reproduction, and the cost of culling animals [2]. Brucellosis in humans causes inconstant fevers, sweating, weakness, anemia, headaches, depression, and muscle and joint pain [3]. If untreated, the disease can localize in the bones and joints, cause discospondylitis of the lumbar spine, or become chronic [4]. Increased rates of premature delivery, spontaneous abortion, and intrauterine infection and fetal death have been described among pregnant women with clinical evidence of brucellosis [5]. In males, orchitis and epididymitis caused by *Brucella* can lead to temporary or permanent infertility [6].

Humans contract brucellosis through direct contact with infected animals and animal exudates or through consumption of contaminated unpasteurized milk and milk products [1], [7]. Identifying risk factors for *Brucella* contamination of raw whole milk has great significance for the prevention and control of human brucellosis. For example, there is a close relationship between *Brucella* contamination of raw milk and the incidence of brucellosis in dairy cows [8]. This represents a significant occupational biohazard to milkers, farmers, laboratory workers and abattoir workers. In addition, raw milk is not only used to make ice cream, cheeses and bakery products [9], but is also supposed to have greater nutritional value and to provide greater health benefits than pasteurized milk and milk powder, and thus it may even be drunk as ready-to-eat food in some countries [10].

Brucellosis frequently presents as a fluctuating animal epidemic [2]. Failure to eradicate it completely in many countries (including China) is largely due to the complexity of conditions in the different countries affected by brucellosis, which creates considerable difficulty for the identification of risk factors [11]. Although the Chinese government has invested significant amounts of money and resources into brucellosis control and prevention, this has not led to successful eradication of this disease [12]. So far, little has been reported on assessing the association between risk factors and *Brucella* contamination of raw milk in China. In this study, a nationwide survey was conducted to identify risk factors for *Brucella* contamination of raw milk in China. These risk factors were then analyzed by logistic regression and the effects of the risk factors were further interpreted to promote appropriate preventive strategies.

## Methods

### Ethics Statement

This investigation was focused on a special type of food material, the raw whole milk, and did not involve using of live animal. No ethics committee specifically approved this procedure. Sampling is permitted by farm owners and households and complied with their routine milking work.

### Study Design and Milk Sample Collection

A multistage, proportional, stratified sampling method was used to obtain a nationally representative sample between June 2009 and October 2011. In China, the 31 province-level administrative regions are divided into seven national administrative zones. For this study, two or three province-level regions were randomly selected from each administrative zone by regional farming scale, with a total of 15 provincial areas selected for sample collection. Then, the dairy production areas of each research region were assigned random numbers, and 25 sampling locations were randomly selected from the 15 administrative regions. A random number generator was used to select cows from each sampling location. A total of 5211 raw whole milk samples were collected from 5211 cows for this study. The milk sample from each animal was collected into a Whirl-Pak bag (Nasco, USA). Fifty ml of milk were drawn into the aseptic sampling bag and stored at 2–8°C, which analysis were performed within 24 hours.

The choice of variables is based on reviewing literatures and academic discussions with a panel of experts from various areas including risk assessment, veterinary public health and epidemiological survey. Each factor was subdivided into 2–4 levels and given different definitions for further research. In this way, variables were determined that may affect exposure to *Brucella* in the whole raw milk, which was tabulated for collect information when the field sampling.

### Polymerase Chain Reaction (PCR) Analysis

The PCR method described by Ning et al. [8] was used to detect *Brucella* antigens in the raw milk samples. This method detects a specific region of the *Brucella* genome, the IS711 element downstream of the alkB gene. The IS711 has been used to assess milk samples in previous studies [13], [14]. The sequences of the oligonucleotide primers used in this study were 5′-GAGAATAAAGCCAACACCCG-3′ and 5′-GATGGACGAAACCCACGAAT-3′. The primers were used to amplify a 317-bp target sequence that included the IS711 region of the *Brucella* genome. The PCR amplicons produced were confirmed as regions of the *Brucella* genome following DNA sequencing by Genome Sequencing Center of Beijing Genomics Institute (Beijing, China) and subsequent entry of the sequence in the BLAST search engine (http://blast.ncbi.nlm.nih.gov/). Serial tenfold dilutions of DNA standards were subjected to evaluate the sensitivity of the method, and the lowest limit of detection was found to be 0.282 pg/µL.

Fifty ml of each milk sample were centrifuged at 12,000 g for 5 min, and 500 µL of sediment was subjected to genomic DNA extraction. DNA extraction of *Brucella* was strictly performed as described by the manufacturer’s instructions (AxyPrep Bacterial Genomic DNA Miniprep Kit; Axygen Biosciences, USA). PCR mixtures were prepared in volumes of 25 µL containing 2.5 U of Easy Taq DNA Polymerase, 1× Easy Taq buffer, 2 mM MgCl_2_, each deoxynucleoside triphosphate 2.5 mM, 40 pmol of each primer, and 100 ng of purified genomic DNA. Polymerase chain reaction was performed in a thermocycler (Bio-Rad Laboratories Inc., Hercules, CA) under the following conditions: 95°C for 3 min for denaturation, 30 cycles of 95°C for 30 s, 58°C for 30 s, 72°C for 30 s, and finally 72°C for 10 min. PCR products were fractionated in a 1.5% (wt/vol) agarose gel containing 1× tris/borate/ethylenediaminetetraacetic acid (EDTA) (TBE; 100 mM Tris-HCl [pH 8.0], 90 mM boric acid, 1 mM disodium EDTA), stained with an ethidium bromide solution (0.5 mg/mL), and visualized under an ultraviolet transilluminator and photographed (Gel Doc XR, Bio-Rad, Hercules, CA, USA). Visible 317-bp bands were considered IS711-positive products compared to the matched normal controls (see Figure S1 for details). Each sample was tested in triplicate.

### Statistical Analysis

Statistical analyses were performed using SPSS 16.0 software (SPSS Inc., Chicago, IL). Data were tested for normality and the percentage of *Brucella-positive* milk samples was associated with different levels of the risk factor using a contingency table to calculate the Chi square statistic. After univariate statistics were generated, the factor in which the percentages of *Brucella-positive* milk samples have significant difference among the interclass levels was determined as positively affect exposure to *Brucella* in the whole raw milk. These factors were further included in a binary logistic regression model (EQ1) [15] to identify independent risk factors.
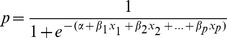
(1)


In this study, the potential risk factors for *Brucella* contamination were categorical variables. Thus, a restricted linear regression containing categorical variables was used to deal with all qualitative objects. That is, when a qualitative variable had *K* possible levels, “*K*−1” independent variables were introduced and treated as “0,1” type variables. In this way, all the data were analyzed by the regression analysis determined by EQ1. Backward stepwise selection (Wald) [16] was used to assess which variables were significant risk factors for *Brucella* contamination of whole raw milk.

After identifying a significant risk factor, it was further decomposed to determine its relative effect on *Brucella* contamination at different levels of the risk factor (*x*). This was calculated as described below. First, the regression coefficient (β_x_) of the risk factor was obtained from the above regression analysis. Second, the relative effects of the *K* different levels of the risk factor were defined as a_1_, a_2_,…a_k_. Third, aimed at the analysis of relative effect, the effect sum of different levels was set as “0”, which meant that the contribution of each level was different within a risk factor, and there was a relative relation in size and positive-negative. Next, the coefficient of each level (excluding the last one) was compare with the last one. That is, the result of comparison would be obtained between each other after finishing successively comparing with a specific value. Finally, the multiple matrix equation group was established (EQ2) for this study to calculate the relative effect of each level in a risk factor.
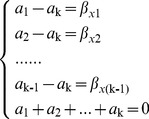
(2)


All p-values were two-tailed and statistical significance was defined as a *P*<0.05.

## Results

In total, 5211 milk samples collected from dairy cattle in 15 Chinese provinces were tested for *Brucella* using PCR, and fifty-six samples (1.07%) were PCR-positive. No PCR-positive samples were obtained from six of the tested provinces, of the remaining nine tested provinces the percentage of positive cattle ranged from 0.23%–3.84%. A comparison of these results with those from international studies is shown in Table 1.

**Table 1:** Comparison of our results with those from other studies.

Location	Positive rate	Number ofpositive samples	Total numberof samples	Detection method	Reference
China	1.07%	56	5211	Polymerase chain reaction	This work
Kenya	0%	0	130	Milk ring test	Namanda AT [32]
Egypt	8.2%	14	170	Milk ring test	El-Kholy AM [33]
Iraq	10.0%	12	120	*Brucella* isolates	Abbas BA [34]
Nigeria	13.5%	27	200	Milk ring test	Bertu WJ [35]
Iran	25.2%	1632	6472	Milk ring test	Zowghi E [36]
Tanzania	55.9%	33	59	Milk ring test	Swai ES [37]

Retrospective analyses of the factors that supposedly affect exposure to *Brucella* found eight factors that significantly differed among interclass levels; these were feeding pattern, herd size, abortion rate, animal polyculture, introduction of new animals, disease prevention and control measures, hygiene, and infection history (Table 2). The different PCR positive rates among the interclass levels are shown in Table 3.

**Table 2:** Different levels and definitions of risk factors for *Brucella* contamination of raw milk.

Factors	Levels	Definitions
Feed pattern (A)	A_1_	Intensive culture, feeding cows and milking by a unified management
	A_2_	Intensive culture, feeding cows and milking managed separately
	A_3_	Scatter breeding, retailing management for milking
Herd size (I)	I_1_	Small farm (20–140)
	I_2_	Medium-sized farm (140–240)
	I_3_	Large farm (>240)
	I_4_	Farming spot (<20)
Abortion rate (J)	J_1_	<2.5%
	J_2_	2.5%–5%
	J_3_	>5%
Animal polyculture (K)	K_1_	No polyculture
	K_2_	Polyculture with small ruminant (goats and sheep)
Introduction of new animals (L)	L_1_	Mating within population, no Introduction
	L_2_	Introduction from abroad
	L_3_	Introduction from domestic
Hygiene* (F)	F_1_	3–4 indexes meet the requirements
	F_2_	2 indexes meet the requirements
	F_3_	0–1 indexes meet the requirements
Disease prevention and control measures (G)	G_1_	Implementing standard vaccine immune by the monitoring policy
	G_2_	Culling after quarantine, no immunization
	G_3_	Nonstandard immunization or culling implementation due to limited conditions
	G_4_	Little immunization and quarantine
Infection history (H)	H_1_	There used to be infected cows in the past
	H_2_	There were not infected cows in the past

* Hygienic indexes by Sanitary Specification for Dairy Farm (Chinese GB16568-2006): 1.environment and facilities; 2.forage; 3.milking; 4.feeding.

**Table 3:** PCR positive rates of *Brucella* among interclass levels of risk factors for *Brucella* contamination of raw milk.

	A	F	G	H	I	J	K	L
1	0.12%	0.04%	0.15%	1.02%	0.10%	0.08%	0.27%	0.23%
2	0.10%	0.17%	0.10%	0.06%	0.02%	0.15%	0.81%	0%
3	0.86%	0.86%	0.83%	–	0.12%	0.84%	–	0.84%
4	–	–	–	–	0.84%	–	–	–

The different levels of each risk factor were quantized by restricted linear regression (Table 4). A total of 5211 records containing 83376 data points formatted by binary quantization were introduced into binary logistic regression analysis to verify the significance of the eight risk factors for *Brucella* contamination. Independent variables were selected by backward stepwise (Wald) method, which means that independent variables were included or excluded from the model according to their Wald statistic. When each variable was assessed independently, not all of the eight risk factors were significant in the logistic regression model. After 12 step iterative operations, 3 factors, *abortion rate (J), animal polyculture (K), and disease prevention and control measures (G)*, were significant in the model (Table 5). These three factors were entered into the model to obtain a logistic regression model for risk factor analysis of *Brucella* contamination in whole raw milk as shown below (EQ3).
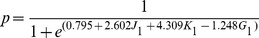
(3)


**Table 4:** Quantization for each level of risk factor.

Factors	Levels	Quantization
Feed pattern (A)	A_1_	A_1_ = 1, A_2_ = 0
	A_2_	A_1_ = 0, A_2_ = 1
	A_3_	A_1_ = 0, A_2_ = 0
Herd size (I)	I_1_	I_1_ = 1,I_2_ = 0,I_3_ = 0
	I_2_	I_1_ = 0,I_2_ = 1,I_3_ = 0
	I_3_	I_1_ = 0,I_2_ = 0,I_3_ = 1
	I_4_	I_1_ = 0,I_2_ = 0,I_3_ = 0
Abortion rate (J)	J_1_	J_1_ = 1,J_2_ = 0
	J_2_	J_1_ = 0,J_2_ = 1
	J_3_	J_1_ = 0,J_2_ = 0
Animal polyculture (K)	K_1_	K_1_ = 1
	K_2_	K_2_ = 0
Introduction of new animals (L)	L_1_	L_1_ = 1,L_2_ = 0
	L_2_	L_1_ = 0,L_2_ = 1
	L_3_	L_1_ = 0,L_2_ = 0
Hygiene (F)	F_1_	F_1_ = 1,F_2_ = 0
	F_2_	F_1_ = 0,F_2_ = 1
	F_3_	F_1_ = 0,F_2_ = 0
Disease prevention and control measures (G)	G_1_	G_1_ = 1,G_2_ = 0,G_3_ = 0
	G_2_	G_1_ = 0,G_2_ = 1,G_3_ = 0
	G_3_	G_1_ = 0,G_2_ = 0,G_3_ = 1
	G_4_	G_1_ = 0,G_2_ = 0,G_3_ = 0
Infection history (H)	H_1_	H_1_ = 1
	H_2_	H_1_ = 0

**Table 5:** Results of logistic regression analyses of risk factors associated with *Brucella* contamination of raw milk.

Factors	Estimate value	Standard error	Wald value	Freedom degree	Significant level
J_1_	−2.602	0.614	17.939	1	0.000
K_1_	−4.309	0.459	88.091	1	0.000
G_1_	1.248	0.562	4.933	1	0.026
Constant	−0.795	0.186	18.283	1	0.000

By using a restricted linear regression containing categorical variables, effect decomposition of significant risk factors in *Brucella* contamination was performed using EQ2. The relative effect for reducing *Brucella* contamination of each level in the risk factor is shown in Table 6.

**Table 6:** Effect decomposition of risk factors to reducing *Brucella* contamination for raw milk.

Factors	Levels	Definitions	Relative effect value
Abortion rate (J)	J_1_	<2.5%	2.91933
	J_2_	2.5%–5%	−0.929667
	J_3_	>5%	−1.989667
Animal polyculture (K)	K_1_	No polyculture	2.1545
	K_2_	Polyculture with small ruminants (goats and sheep)	−2.1545
Disease prevention and control measures (G)	G_1_	Implementing standard vaccine immune by the monitoring policy	2.2165
	G_2_	Culling after quarantine, no immunization	2.9085
	G_3_	Nonstandard immunization or culling implementation due to limited conditions	−1.8856
	G_4_	Little immunization and quarantine	−3.2394

## Discussion

This is the first nationwide study in China to address the factors contributing to *Brucella* contamination of raw whole milk. Because most developed countries are officially free of brucellosis, there is little information available about *Brucella* contamination of raw milk from developed countries [11]. We compared the results of our study with reports from developing countries (Table 1), and found a low risk of contamination in China relative to these other nations.

However, the regional contamination is concerning and begs the following question: which factors exactly are predisposing to *Brucella* infection in Chinese dairy herds? In this study, we identified three independent risk factors*–abortion rate (J), animal polyculture (K), and disease prevention and control measures (G)–*that were specifically associated with *Brucella* contamination. The probability of *Brucella* contamination (*P* value) was reduced when “J_1_ = 1”, “K_1_ = 1” and “G_1_ = 0” were each substituted into EQ3. Further analysis combining the results of effect decomposition, this study shows that the following three conditions could effectively reduce the risk of *Brucella* contamination of raw milk in China. These are:
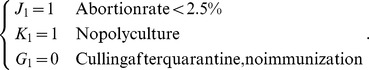



Abortion is one of the major clinical results of brucellosis. Not only does pregnancy loss in dairy cows produce large economic, breeding and production losses for the dairy industry [17], but aborted fetuses, placentas and secretions are also one of the most infective sources of *Brucella* [18]. Relative to the fever symptoms caused by *Brucella*, abortion is easily observed and is often used to evaluate vaccine efficacy, as well as being one factor used for assessment of infection risk. In infected dairy cattle, *Brucella* is localized in the tissues of the udder and is excreted along with milk even after abortion. [19]. It was not surprising that abortion rate (J) was a significant risk factor by our logistic regression analysis in this study. The results of effect decomposition showed that the relative effect to control risk was reduced along with the increase in the abortion rate. This means that raw whole milk is at high-risk for *Brucella* contamination when the abortion rate is at a fairly high level in the herd.

Animal polyculture (K) remained in the final logistic model as one of the significant risk factors, and the results of effect decomposition distinctly showed that contact with small ruminants (goats and sheep) would increase the probability of *Brucella* contamination of whole milk. Although it is well known that *Brucella* is easily transmitted among domesticated animals, such as cattle, goats, sheep, pigs, and buffalo [20], assessing the amount of close contact with other domesticated animal species is often ignored in the implementation of prevention and control strategies. Most efforts have been focused on reducing *Brucella* infection in dairy herds; however, goat and sheep flocks infected with brucellosis have been widely reported worldwide, and the infection rates of these species are second only to that of cattle [2], [21]. Notably, an increase in *Brucella* infection has been recently confirmed in sheep and goat flocks in China [11], [22]. As the main product of the Chinese dairy market, cow milk has been closely monitored by the Chinese government for the past few years. However, goat milk is often consumed by private individuals as a substitute milk product, and there has been little monitoring of goat milk. In this epidemiologic investigation, we noticed the worrying phenomenon that cows and goats are raised together in polyculture on some family farms because of their similar husbandry requirements. Statistical tests showed that there was a significant difference (*P*<0.05) in the number of PCR-positive samples from cows living in polyculture with goats and the number of PCR-positive samples from those that do not (Table 3). The verification of the models showed that polyculture was one of the significant risk factors, which indicates that infected small ruminants are a potential source of brucellosis infection for dairy cows.

The relative effect assessment of risk factor (G), *disease prevention and control measures*, presents a significant conclusion in this study (Table 6). Previously, the question of whether vaccination or culling is a better strategy for controlling brucellosis has long been debated among academics and in government circles. As an economic control measure for this disease, the *B. abortus* strain 19 vaccine was introduced for field use in the USA in 1941 [23] and in China in 1958 [11]. Several *Brucella* vaccines have been used in China for prevention and control of brucellosis in the past decades [11]; however, some drawbacks of the vaccine, including low quality and interference of vaccine-related antibodies with diagnostic testing, have long plagued researchers and policymakers. Although these problems have been well resolved with the Strain RB51 vaccine, which has the advantage of producing protection apparently comparable to Strain 19 without inducing the titers that cause diagnostic confusion, administration of the currently available vaccine alone is not sufficient for the eradication of brucellosis in any country [24].

Efforts to eradicate brucellosis caused by *Brucella abortus* have been successful in some countries [18]. All US states were declared free of brucellosis in cattle in 2007 through firm implementation of the State-Federal Brucellosis Eradication Program [25]. A chief factor in the success of the program was the acceptance of eradication program procedures by livestock owners. Inevitably, in addition to the inconvenience and work required, an eradication program will reduce the cattle population and cause economic losses. This has made it difficult to implement culling procedures, especially in developing countries (including China), although the government has often provided considerable funds to subsidize farmers’ losses. In the past, there was no definitive answer as to whether vaccination or culling was the best option for China. The results of this study, obtained by regression analysis of a large sample, support the implementation of an eradication program in China. Although the use of vaccination has been a major factor in the success of the brucellosis eradication program, it also makes it complicated to monitor for the disease. Establishing an eradication program in China is a highly complex proposition, and persistent implementation of an eradication policy seems the most likely means of achieving eradication of the disease.

Feeding pattern (A) and herd size (I) have been thought to be important determinants of herd infection dynamics [26]. There is some evidence suggesting that if the disease is introduced into larger herds, a higher proportion of the animals in the herd become infected and the disease persists and is more difficult to eradicate [27], [28]. However, the results of this study suggest that feeding pattern or herd size is less important a risk factor in the Chinese dairy industry than the three factors discussed previously. Implementing a strict eradication program, avoiding polyculture, and maintaining a low abortion rate are the interventions most likely to reduce the brucellosis infection rate. Further model analysis indicates that raw milk has a low risk of *Brucella* contamination as long as these three risk factors are well controlled in herds of any size.

Uncontrolled animal transportation through “open” borders increased the risk that brucellosis would spread in some regions. Purchasing animals and artificial insemination have both been identified as risk factors for brucellosis [29]. However, although it is an important aspect of brucellosis control, introduction of new animals (L) does not appear as one of the final significant risk factors in this study. This is because of the strict management measures of the Chinese government and increased farmer awareness of brucellosis prevention and control. Owners have consciously introduced a high-quality bull into their herd, or increased disease monitoring before breeding, to avoid the emergence of cross-infection.

Poor environmental hygiene is a risk factor for many diseases. Fortunately, *Brucella* contamination of milk is not usually through environmental contamination. That may partly explain why hygiene (F) was not one of the final significant risk factors in our model. As a facultative intracellular pathogen, *Brucella* can localize to the mammary glands after cattle are infected [30], and bacteria present in the lymph nodes may be intermittently shed into milk. Therefore, endogenous source pollution is considered the main route of *Brucella* contamination of raw whole milk. A history of brucellosis infection (H) in a herd is also often considered a risk factor in epidemiological surveys. However, the results of this study imply that the negative effects of a brucellosis infection history can be counteracted by implementing a strict eradication program, avoiding polyculture, and maintaining a low abortion rate.

Without a reliable interpretation of the numbers generated by the models, the results are meaningless and cannot be taken into account in epidemiologic studies or when implementing new policies, no matter how elaborate or thoughtful the statistical modeling [31]. Brucellosis causes economic losses and is a human health hazard; thus, it should not be ignored. Control of infectious diseases must start by addressing the three aspects of infectious disease transmission: reservoir of infection, route of transmission and susceptible populations. This study reveals the possible risk factors and helps in designing highly efficient prevention strategies for decreasing the risk of *Brucella* contamination of raw whole milk in China. In summary, the risk of *Brucella* contamination of raw whole milk would be significantly reduced by removing an infectious reservoir by avoiding animal polyculture, cutting off transmission routes by establishing an eradication program, and protecting the susceptible by maintaining a low abortion rate.

## Supporting Information

Figure S1 Agarose gel electrophoresis of PCR assay for detection of *Brucella* DNA. Lane M: molecular weight marker; Lane 1: Polymerase chain reaction (PCR)-positive control (*Brucella suis* strain 2); Lane 2–5: PCR products amplified from *Brucella* DNA extracted from part of raw milk samples; Lane 6: PCR-negative control (no DNA).
(DOCX)

## References

[1] Corbel MJ (1997) Brucellosis: an overview. Emerg Infect Dis 3: 213–221. 9204307 10.3201/eid0302.970219 PMC2627605

[2] Gwida M, Dahouk SA, Melzer F, Rösler U, Neubauer H, et al (2010) Brucellosis–Regionally emerging zoonotic disease? Croat Med J 51: 289–295. 20718081 10.3325/cmj.2010.51.289 PMC2931433

[3] Dossey BM (2010) Florence Nightingale: Her Crimean Fever and Chronic Illness. J Holist Nurs 28(1): 38–53. 20467026 10.1177/0898010109356472

[4] Mantur BG, Amarnath SK, Shinde RS (2007) Review of clinical and laboratory features of human brucellosis. J Med Microbiol 25: 188–202. 10.4103/0255-0857.34758 17901634

[5] Khan MY, Mah MW, Memish ZA (2001) Brucellosis in Pregnant Women. Clin Infect Dis 32: 1172–1177. 11283806 10.1086/319758

[6] Navarro-Martínez A, Solera J, Corredoira J, Beato JL, Martínez-Alfaro E, et al (2001) Epididymoorchitis Due to *Brucella* mellitensis: A Retrospective Study of 59 Patients. Clin Infect Dis 33: 2017–2022. 11698991 10.1086/324489

[7] Earhart K, Vafakolov S, Yarmohamedova N, Michael A, Tjaden J, et al (2009) Risk factors for brucellosis in Samarqand Oblast, Uzbekistan. Int J Infect Dis 13: 749–753. 19457689 10.1016/j.ijid.2009.02.014

[8] Ning P, Guo K, Xu L, Xu R, Zhang C, et al (2012) Evaluation of Brucella infection of cows by PCR detection of *Brucella* DNA in raw milk. J.Dairy Sci 95: 4863–4867. 22916890 10.3168/jds.2012-5600

[9] Brooks JC, Martinez B, Stratton J, Bianchini A, Krokstrorn R, et al (2012) Survey of raw milk cheeses for microbiological quality and prevalence of foodborne pathogens. Food Microbiol 31: 154–158. 22608218 10.1016/j.fm.2012.03.013

[10] LeJeune JT, Rajala-Schultz PJ (2009) Unpasteurized Milk:A Continued Public Health Threat. Food Safety 48: 93–100. 10.1086/595007 19053805

[11] Pappas G, Papadimitriou P, Akritidis N, Christou L, Tsianos EV (2006) The new global map of human brucellosis. Lancet Infect Dis 6: 91–99. 16439329 10.1016/S1473-3099(06)70382-6

[12] Shang DQ, Xiao DL, Yin JM (2002) Epidemiology and control of brucellosis in China. Vet microbiol 90: 165–182. 12414142 10.1016/s0378-1135(02)00252-3

[13] Hamdy MER, Amin AS (2002) Detection of *Brucella* species in the milk of infected cattle, sheep, goats and camels by PCR. Vet J 163: 299–305. 12090772 10.1053/tvjl.2001.0681

[14] O’Leary S, Sheahan M, Sweeney T (2006) *Brucella abortus* detection by PCR assay in blood, milk and lymph tissue of serologically positive cows. Res. Vet Sci 81: 170–176. 10.1016/j.rvsc.2005.12.001 16545848

[15] Dayton CM (1992) Logistic regression analysis. Stat, 474–574.

[16] Steyerberg EW, Eijkemans MJC, Habbema JDF (1999) Stepwise selection in small data sets: a simulation study of bias in logistic regression analysis. J Clin Epidemiol 52(10): 935–942. 10513756 10.1016/s0895-4356(99)00103-1

[17] Rafati N, Mehrabani-Yeganeh H, Hanson TE (2010) Risk factors for abortion in dairy cows from commercial Holstein dairy herds in the Tehran region. Prev Vet Med 96: 170–178. 20598387 10.1016/j.prevetmed.2010.05.008

[18] Francisco J, Vargas O (2002) Brucellosis in Venezuela. Vet Microbiol 90: 39–44. 12414132 10.1016/s0378-1135(02)00243-2

[19] Cordes DO, Carter ME (1979) Persistency of *Brucella abortus* infection in six herds of cattle under brucellosis eradication. New Zeal Vet J 27: 255–259. 10.1080/00480169.1979.34666 119934

[20] Corbel MJ (2006) Brucellosis in Humans and Animals. World Health Organization, Geneva, Switzerland.

[21] Mohamed NS, Boyle SM, Sriranganathan N (2010) Brucellosis: A re-emerging zoonosis. Vet Microbiol 140: 392–398. 19604656 10.1016/j.vetmic.2009.06.021

[22] Qiu JL, Wang WJ, Wu JB, Zhang H, Wang YZ, et al (2012) Characterization of Periplasmic Protein BP26 Epitopes of Brucella melitensis Reacting with Murine Monoclonal and Sheep Antibodies. PloS one 7(3): e34246. 22457830 10.1371/journal.pone.0034246 PMC3311636

[23] Olsen SC, Stoffregen WS (2005) Essential role of vaccines in brucellosis control and eradication programs for livestock. Expert Rev Vaccines 4(6): 915–928. 16372886 10.1586/14760584.4.6.915

[24] Oliveira SC, Giambartolomei GH, Cassataro J (2011) Confronting the barriers to develop novel vaccines against brucellosis. Expert Rev Vaccines 10(9): 1291–1305. 21919619 10.1586/erv.11.110

[25] Ragan VE (2002) The Animal and Plant Health Inspection Service (APHIS) brucellosis eradication program in the United States. Vet Microbiol 90: 11–18. 12414129 10.1016/s0378-1135(02)00240-7

[26] Salman MD, Meyer ME (1984) Epidemiology of bovine brucellosis in the Mexicali Valley, Mexico: literature review of disease-associated factors. Am J Vet Res 45(8): 1557–1560. 6476569

[27] Omer MK, Skjerve E, Woldehiwet Z, Holstad G (2000) Risk factors for *Brucella spp.* infection in dairy cattle farms in Asmara, State of Eritrea. Prev Vet Med 46: 257–265. 10960712 10.1016/s0167-5877(00)00152-5

[28] Muma JB, Samui KL, Oloya J, Munyeme M, Skjerve E (2007) Risk factors for brucellosis in indigenous cattle reared in livestock-wildlife interface areas of Zambia. Prev Vet Med 80: 306–317. 17481753 10.1016/j.prevetmed.2007.03.003

[29] Stringer LA, Guitian FJ, Abernethy DA, Honhold NH, Menzies FD (2008) Risk associated with animals moved from herds infected with brucellosis in Northern Ireland. Prev Vet Med 84: 72–84. 18207262 10.1016/j.prevetmed.2007.11.005

[30] Xavier MN, Paixão TA, Poester EP, Lage AP, Santos RL (2009) Pathological Immunohistochemical and Bacteriological Study of Tissues and Milk of Cows and Fetuses Experimentally Infected with Brucella abortus. J Comp Pathol 140: 149–157. 19111839 10.1016/j.jcpa.2008.10.004

[31] Kaufman JS, Hernán MA (2012) Epidemiologic Methods Are Useless: They Can Only Give You Answers. Epidemiology 23(6): 785–786. 23038106 10.1097/EDE.0b013e31826c30e6

[32] Namanda AT, Kakai R, Otsyula M (2009) The role of unpasteurized “hawked” milk in the transmission of brucellosis in Eldoret municipality, Kenya. J Infect Developing Countries 3(4): 260–266. 10.3855/jidc.122 19759488

[33] El-Kholy AM, Meshref AMS, Hassan GM (2008) Sero-diagnosis of brucellosis in cows by using milk. Assiut Vet Med J 54: 117.

[34] Abbas BA, Aldeewan AB (2009) Occurrence and epidemiology of *Brucella* spp. in raw milk samples at Basrah province, Iraq. Bulg. J Vet Med 12(2): 136–142.

[35] Bertu WJ, Dapar M, Gusi1 AM, Ngulukun SS, Leo S, et al (2010) Prevalence of brucella antibodies in marketed milk in Jos and environs. Afr J Food Sci 4(2): 062–064.

[36] Zowghi E, Ebadi A, Mohseni B (1990) Isolation of *Brucella* organisms from the milk of seronegative cows. Rev Sci Tech: 1175–1178. 10.20506/rst.9.4.525 2132709

[37] Swai ES, Schoonman L (2011) Microbial quality and associated health risks of raw milk marketed in the Tanga region of Tanzania. Asian Pac J Trop Bed: 217–222. 10.1016/S2221-1691(11)60030-0 PMC3609189 23569762

