# Supplementary material for: Identification and Effect Decomposition of Risk Factors for Brucella Contamination of Raw Whole Milk in China
Source: PLoS One. 2013 Jul 10;8(7):e68230. doi: 10.1371/journal.pone.0068230 (PMC3707899; doi:10.1371/journal.pone.0068230)
Supplement: Figure S1 — Agarose gel electrophoresis of PCR assay for detection of Brucella DNA. Lane M: molecular weight marker; Lane 1: Polymerase chain reaction (PCR)-positive control (Brucella suis strain 2); Lane 2–5: PCR products amplified from Brucella DNA extracted from part of raw milk samples; Lane 6: PCR-negative control (no DNA). (DOCX) [file pone.0068230.s001.docx]

*Research Article*

**Identification and effect decomposition of risk factors for *Brucella* contamination of raw whole milk in China**

Pengbo Ning^a^ , Mancai Guo^b^, Kangkang Guo^a^, Lei Xu^a^, Min Ren^a^, Yuanyuan Cheng^a^ & Yanming Zhang^a,^^[[1]](#footnote-1)^*

^a^ *College of Veterinary Medicine, Northwest A&F University, Yangling, Shaanxi 712100, PR China*

^b^ *College of Science, Northwest A&F University, Yangling, Shaanxi 712100, PR China*

SUPPLEMENTARY MATERIAL

Supplementary Figure S1. Agarose gel electrophoresis of PCR assay for detection of *Brucella* DNA. Lane M: molecular weight marker; Lane 1: Polymerase chain reaction (PCR)-positive control (*Brucella suis* strain 2); Lane 2-5: PCR products amplified from *Brucella* DNA extracted from part of raw milk samples; Lane 6: PCR-negative control (no DNA).


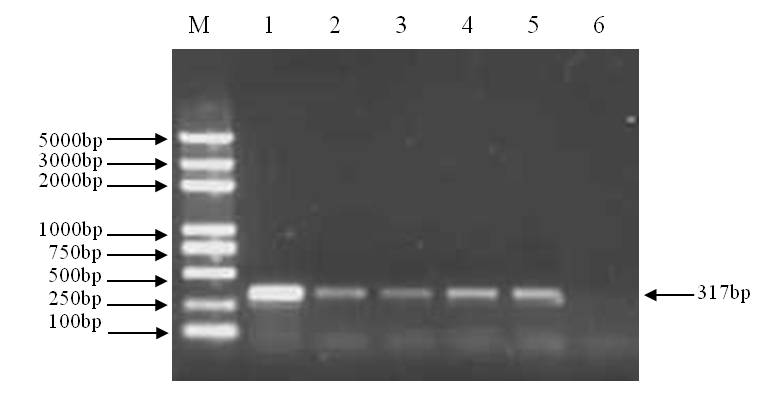


1. * Corresponding author，Tel.: +86 29 8709 2040, Fax 86 29 8709 1032.

   *E-mail addre*ss: [tommy0627@163.com](mailto:tommy0627@163.com) (Pengbo Ning); zhangym@nwsuaf.edu.cn (Yanming Zhang) [↑](#footnote-ref-1)
